# The genomic landscape associated with resistance to aromatase inhibitors in breast cancer

**DOI:** 10.5808/gi.23012

**Published:** 2023-06-30

**Authors:** Kirithika Sadasivam, Jeevitha Priya Manoharan, Hema Palanisamy, Subramanian Vidyalakshmi

**Affiliations:** Department of Biotechnology, PSG College of Technology, Coimbatore 641004, Tamil Nadu, India

**Keywords:** acquired drug resistance, ER-positive breast cancer, non-steroidal Ais, pathway analysis, TCGA-BRCA patient data

## Abstract

Aromatase inhibitors (AI) are drugs that are widely used in treating estrogen receptor (ER)–positive breast cancer patients. Drug resistance is a major obstacle to aromatase inhibition therapy. There are diverse reasons behind acquired AI resistance. This study aims at identifying the plausible cause of acquired AI resistance in patients administered with non-steroidal AIs (anastrozole and letrozole). We used genomic, transcriptomic, epigenetic, and mutation data of breast invasive carcinoma from The Cancer Genomic Atlas database. The data was then separated into sensitive and resistant sets based on patients’ responsiveness to the non-steroidal AIs. A sensitive set of 150 patients and a resistant set of 172 patients were included for the study. These data were collectively analyzed to probe into the factors that might be responsible for AI resistance. We identified 17 differentially regulated genes (DEGs) among the two groups. Then, methylation, mutation, miRNA, copy number variation, and pathway analyses were performed for these DEGs. The top mutated genes (*FGFR3, CDKN2A, RNF208, MAPK4, MAPK15, HSD3B1, CRYBB2, CDC20B, TP53TG5*, and *MAPK8IP3*) were predicted. We also identified a key miRNA - hsa-mir-1264 regulating the expression of CDC20B. Pathway analysis revealed HSD3B1 to be involved in estrogen biosynthesis. This study reveals the involvement of key genes that might be associated with the development of AI resistance in ER-positive breast cancers and hence may act as a potential prognostic and diagnostic biomarker for these patients.

## Introduction

According to the World Health Organization (2020), about 2.3 million women were diagnosed with breast cancer around the world, and approximately 685,000 individuals are currently deceased [1]. There are different types of breast cancer, out of which 75% are hormone receptor–positive (HR-positive) breast cancer [2], that may be either estrogen receptor–positive (ER-positive) or progesterone receptor–positive. Most females across the world suffer from ER-positive breast cancer during their post-menopausal and pre-menopausal stages [3].

ER-positive breast cancer cells contain receptors for estrogen, which upon binding to the hormone help in the proliferation of cancer. This information is vital in deciding the treatment method for ER-positive breast cancer [4]. The existing breast cancer treatment targets the inhibition of estrogen and the prevention of hormone-receptor binding using ER modulators (tamoxifen), inhibitors (aromatase inhibitors), and ER degrading agents like fulvestrant [5,6]. Generally, combinatorial therapy is preferred which comprises surgery, endocrine therapy, chemotherapy, immunotherapy, etc. During such multiple treatments, aromatase inhibitors (AIs) are often administered to both pre and postmenopausal women as part of the endocrine therapy. Currently, AIs are reported to be one of the efficient drugs that are used in treating ER-positive breast cancer [7,8].

Aromatase inhibitors are drugs that inhibit the enzyme aromatase [9]. It is responsible for catalyzing the conversion of testosterone and androstenedione to estradiol and estrone, the rate-limiting step in estrogen biosynthesis [10]. Estradiol and estrone are the biologically active forms of estrogen detected in non-pregnant women. Researchers realized the need to target aromatase enzymes to improve ER-positive breast cancer treatment and started the development of AIs in the early 1970s [11]. Currently, there are three generations of AIs named in the chronological order of discovery. Aminoglutethimide is one of the well-known first-generation AIs. Fadrozole and rogletimide belonging to the second-generation AIs were reported to be efficient in treating breast cancer with few undesirable pharmacokinetic properties [12]. The frequently used drugs like anastrozole (Arimidex), letrozole (Femara), and Exemestane (Aromasin) fall under the third generation of AIs [13,14]. The third generations of AIs are further classified based on their mechanism of action and chemical structure into steroidal (type 1) and non-steroidal (type 2) AIs [15-17]. Steroidal AIs such as Aromasin (third generation AIs) compete with the substrate of aromatase and bind covalently to the active sites resulting in an irreversible inhibition [18]. Whereas, non-steroidal AIs (anastrozole and letrozole) bind non-covalently to the heme moiety of the enzyme resulting in a reversible inhibition [19].

Even though AIs are very effective in treating breast cancer, drug resistance seems to be a major problem while administering them. There are two types of resistance that can be observed in patients, namely *de novo*/primary resistance and acquired drug resistance [15]. When a patient does not respond to the initial administration of the drug, then the individual possesses *de novo*/primary drug resistance. Whereas, when the patient is sensitive to the drug initially and later develops resistance after 2–3 years, it is categorized as an acquired drug resistance. Patients with acquired drug resistance often relapse. The current strategy that is being used to treat AI-resistant patients is to change the type of drug that is being administered to them [20-22]. For example, if a patient being treated with Aromasin develops acquired resistance, then the individual is continued with the administration of another AI like anastrozole. The development of acquired drug resistance is intricate with a complex interplay of multiple genetic profiles and hence, the exact mechanism of drug resistance must be explored. This would help in planning for better management of ER-positive breast cancer.

We aim to determine the plausible genetic and epigenetic factors that might play a vital role in the development of non-steroidal AI resistance. The breast cancer patients treated with non-steroidal drugs like anastrozole and letrozole were chosen for this analysis from the The Cancer Genome Atlas (TCGA) database. Complete genomic, transcriptomic, and epigenomic analyses were performed with this data set for the resistant and sensitive groups and compared to identify the underlying mechanism of drug resistance.

## Methods

**Fig. 1.**
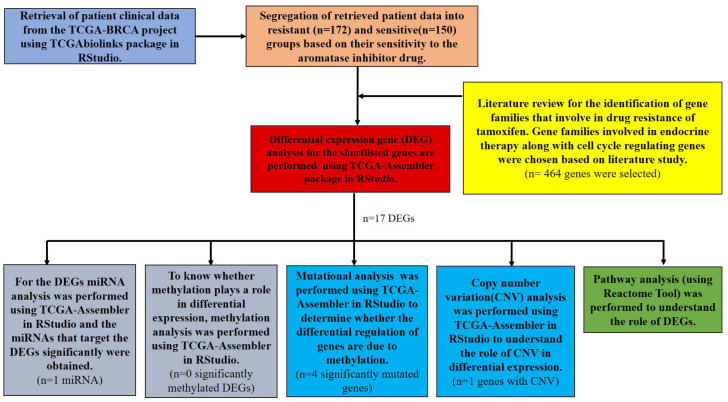
Outline of the computational analysis performed for identifying the genomic landscape for non-steroidal aromatase inhibitors resistance in breast cancer. TCGA, The Cancer Genome Atlas.

NCI’S Genomic Data Common (GDC) data portal was used to access TCGA project (https://portal.gdc.cancer.gov/). The Breast Invasive Carcinoma project (TCGA-BRCA) was chosen from the list of TCGA projects for this study. The entire study design is shown in Fig. 1.

### Clinical data retrieval

Clinical data of patients administered with anastrozole and letrozole were downloaded from the TCGA database, using the TCGAbiolinks package in RStudio [23]. The retrieved sample data were further classified into resistant and sensitive groups based on the patient’s responsiveness to the drug and recurrence of the tumor. The sensitive group is defined by the patient showing response to the drugs after 2 years of initial treatment [24] and the individual not reporting a relapse. The resistant group is categorized as the lack of patients’ response to the drug after initial treatment and also shows signs of cancer relapse.

### Transcriptomic analysis

#### Identification of differentially expressed genes by gene expression analysis

Processed RNA-sequencing data were retrieved from the TCGA database using TCGA-Assembler in RStudio [265]. A total of 464 genes were chosen for analysis from an exhaustive literature analysis [20,26-33]. The significant (p<0.05) differentially expressed genes (DEGs) with <0.5 log 2-fold change and >–0.5 log 2-fold change were identified and a volcano plot was constructed to represent the data.

#### Methylation data analysis

For methylation analysis, corresponding probes for differentially expressed genes were taken from TCGA-Illumina human DNA methylation 450K platform. Probes having differential beta values were calculated by subtracting the average beta value of each probe in the sensitive sample from the average beta value of the same in resistant samples. Probes having an absolute beta value difference of more than 0.2 and less than –0.2 with a significant p-value (p ≤ 0.05) were considered to be hypomethylated or hypermethylated probes respectively.

#### miRNA analysis of the DEGs: miRNA analysis

Using the expression data available for miRNA for the sensitive and resistant samples in the TCGA dataset, the fold change of each miRNA in the above-fetched genes were obtained. Then, miRNAs that were differentially expressed in the TCGA dataset were subjected to analysis of both validated and predicted gene targets in the Mirwalk database (http://mirwalk.umm.uni-heidelberg.de/) [34]. These gene targets were then verified using the miRDB (http://mirdb.org/cgi-bin/search.cgi) [35], and the Targetscan databases (http://www.targetscan.org/vert_80/) [36]. From this analysis, a list of miRNAs targeting certain differentially expressed genes were obtained. The miRNA which was reciprocally regulated as compared to their respective gene expression patterns were represented in the results.

### Genomic analysis

#### Mutational analysis

Mutation data of the resistant and sensitive sets were also downloaded using TCGA-Assembler in R Studio from the TCGA database. The retrieved data was then filtered and the top 10 mutated genes were identified by plotting a Plotmaf summary in RStudio using the TCGAbiolinks package. A Venn diagram showing the mutated genes in sensitive and resistant data sets was plotted. To identify the percentage of mutation within the sensitive and resistant sets, an oncoplot was plotted for the top mutated genes using ggplot2 package (version 3.3.5). Further, lollipop (g3viz package- version 1.1.4) plots were plotted for the identified top mutated genes that were unique in the resistant set using g3viz package (version 1.1.4).

#### Copy number variation analysis of the DEGs

The TCGA-Assembler was used to download the copy number variation data of the identified DEGs from the TCGA database. The Genomic Analysis of Important Aberrations (GAIA) plots were drawn for the processed copy number variation (CNV) in RStudio using GAIA package version 2.36.0.

### Protein-protein interaction network

A protein-protein interaction network (PPI) was constructed for the DEGs (17) using the STRING database (https://string-db.org/) [37] with a minimum confidence of 0.150. The genes in the built network were then clustered by the K-means clustering module of the STRING server.

### Pathway analysis of DEGs

Pathway enrichment of the identified DEGs was performed using the Reactome browser (https://reactome.org/) [38,39]. Significant pathways of the DEGs were explored and the predominant genes involved in estrogen biosynthesis were identified.

## Results

### Genetic and epigenetic alterations of DEGs involved in resistance to non-steroidal aromatase in breast cancer

**Fig. 2.**
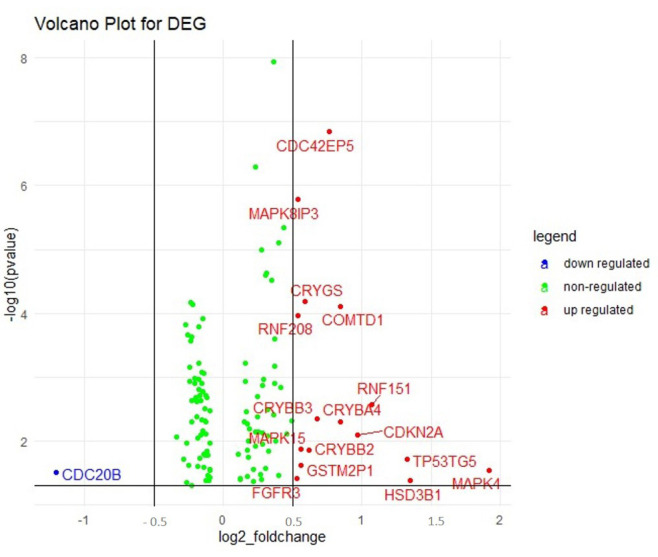
Volcano plot displaying the differentially regulated genes (DEGs) obtained in gene expression analysis. The upregulated (16) and the downregulated genes (1) are represented in red and blue dots respectively. The green dots indicated the non-regulated genes in the array.

A total of 322 patient barcodes were retrieved from the TCGA-BRCA project (TCGA database). Among the 322 patient barcodes, 150 patient barcodes were segregated as sensitive and 172 as resistant categories and were used for further analyses. We identified the DEGs among the resistant and sensitive categories. It was found that 121 genes were significantly expressed, of which 16 genes were up-regulated and 1 gene was down-regulated (Fig. 2, Supplementary Table 1).

The epigenetic mechanisms behind differential regulation were analyzed for the DEGs. Differential methylation analysis indicated that the DEGs were not subjected to epigenetic alterations.

To understand the role of miRNAs in the regulation of the differentially expressed genes, Mirwalk was used to analyze the predicted and validated miRNAs targeting these genes. In our analyses, miRNAs that were upregulated in the TCGA dataset targeting downregulated genes and vice versa were focused on. Using the TCGA expression dataset for miRNA and mRNA, it was found that a total of 20 miRNAs were differentially regulated, 19 miRNAs being up-regulated and 1 miRNA being down-regulated. The gene CDC20B and miRNA hsa-mir-1264 were found to obey the reciprocal rule between the target gene and miRNA expression.

### Genetic mutations of DEGs that might drive non-steroidal aromatase resistance in breast cancer

**Fig. 3.**
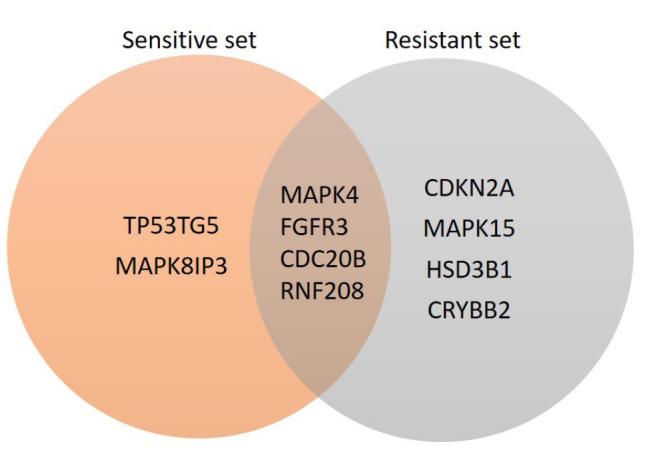
Venn diagram for mutational analysis of differentially regulated genes. The genes that are uncommon between the sensitive and resistant set are represented.

To investigate mutations in the DEGs identified in this study, we analyzed the mutations in these genes in the whole-exome sequencing (WES) data of breast cancer samples from TCGA. Analysis of TCGA WES data of the samples indicated that there were eight genes significantly mutated in the resistant samples and six genes significantly mutated in the sensitive samples. The results obtained from the analysis are depicted as a Venn diagram shown in Fig. 3. The genes *TP53TG5* and *MAPK8IP3* were distinctively mutated in the sensitive set (Fig. 3). Similarly, the genes *CDKN2A, MAPK15, HSD3B1*, and *CRYBB2* were distinctively seen to be mutated in the patients showing resistance (Fig. 3). These unique genes (not mutated commonly in both groups) are proposed to influence the drug resistance mechanism. Hence further analyses were carried out with these unique genes.

**Fig. 4.**
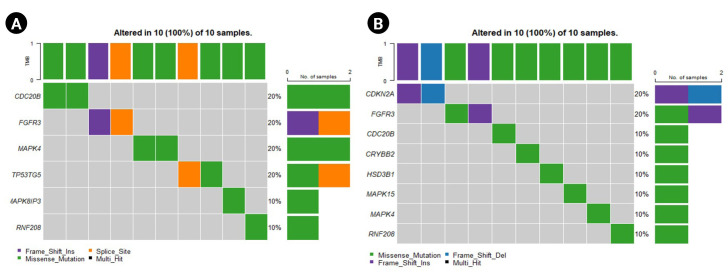
Oncoplots for the sensitive and resistant barcodes of the genes that are predominantly mutated in the population. (A) Sensitive group. (B) Resistant group.

The oncoplot shows the genes that are highly mutated in the sensitive and resistant set (Fig. 4A and 4B). The plot indicates the percentage population possessing the mutation along with the type of mutation for the corresponding gene. Fig. 4 represents the predominantly mutated genes in the population. The unique genes reported above are also seen to be predominantly mutated in both the sensitive and the resistant sets. Genes such as *TP53TG5* and *MAPK8IP3* are present in the top mutated genes in the oncoplot for the sensitive set (Fig. 4A). Similarly, *CDKN2A, MAPK15, HSD3B21*, and *CRYBB2* are present among the top mutated genes in the oncoplot plotted for the resistant set (Fig. 4B).

Further, the lollipop plots reveal the location of the mutation present in these unique genes (Supplementary Table 2). Supplementary Fig. 1 shows the location of mutations in the unique genes observed in the sensitive samples and Supplementary Fig. 2 for the resistant samples. A missense mutation was seen in the *TP53TG5* gene outside the TP53IP5 domain region, indicating an alteration in its function (Supplementary Fig. 1A). Additionally, a missense mutation was seen in the *MAPK8IP3* gene at the PARP domain (Supplementary Fig. 1B).

A frameshift deletion and a frameshift insertion mutation were observed in the gene *CDKN2A* (Supplementary Fig. 2A). A missense mutation was noticed after 200 bp in the *CRYBB2* gene (Supplementary Fig. 2B). The domain 3-beta HSD of the *HSD3B1* gene tended to have a missense mutation in the resistant group (Supplementary Fig. 2C). Similarly, a missense mutation was noted at 544 bp of the *MAPK15* gene (Supplementary Fig. 2D).

#### CNV analysis

**Fig. 5.**
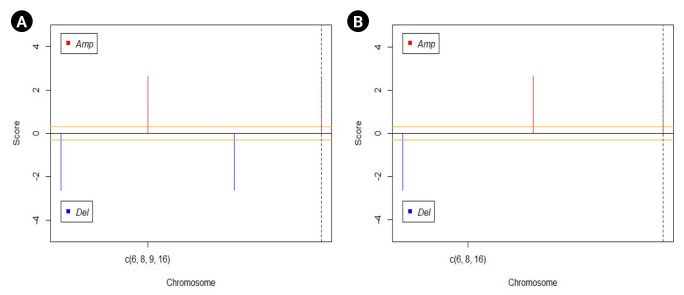
Genomic Analysis of Important Aberrations (GAIA) plots drawn for visualization of variation in differentially regulated genes. (A) Sensitive dataset. (B) Resistant dataset.

The GAIA plots obtained for the CNV data of the two groups help us in visualizing the variation in the DEGs. Fig. 5 represents the GAIA plots of sensitive and resistant groups. The plots reveal that the genes *MAPK15* and *MAPK8IP3* located in chromosomes 8 and 16 respectively were amplified in both sensitive and resistant samples. It was also seen that the gene *GSTM2P1* located in chromosome 6 was deleted in both the sample sets, whereas the gene *CDK2NA* present on chromosome 9 was deleted only in the sensitive set.

### Protein-protein interaction of the DEGs

The PPI network of the DEGs was built with 16 nodes and 17 edges with a medium confidence score of 0.150 and an enriched p-value of 0.000478 (Supplementary Fig. 3) using the STRING server.

**Fig. 6.**
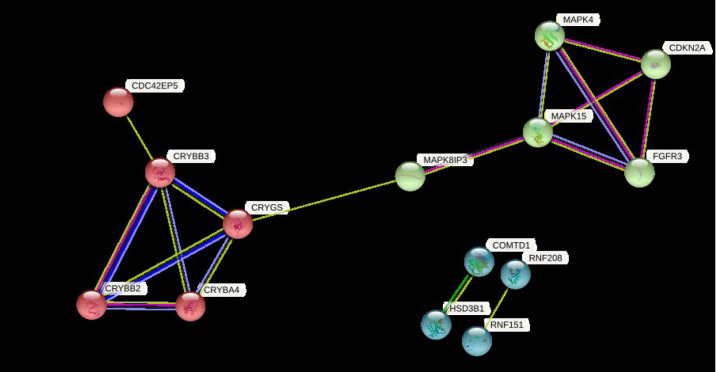
Clustering of differentially regulated genes by STRING database to visualize the genes that are similar.

Clustering of DEGs resulted in three clusters with an average local clustering coefficient of 0.719 (Fig. 6). The top cluster (cluster 1) comprised the proteins MAPK4, CDKN2A, MAPK15, FGFR3, and MAPK8IP3. Whereas cluster 2 and cluster 3 included CRYBB2, CRYBA4, CDC42EP5, CRYGS, CRYBB3 and COMTD1, HSD3B1, RNF151, RNF208, respectively.

### Pathway analysis of DEGs

The significant pathways associated with the DEGs were explored using the Reactome tool. The DEGs (17) were enriched in 105 pathways (data not shown), where 7 pathways (Table 1) corresponded to estrogen biosynthesis. The remaining pathways of DEGs were related to cancer, disease, cell cycle, signaling, and other regulating pathways. From the pathway analysis, it was observed that the gene *HSD3B1* was indirectly involved with estrogen biosynthesis.

## Discussion

AI drug resistance is one of the major problems caused while treating ER-positive breast cancer. Identification of a significant biomarker for predicting non-steroidal AI drug resistance will help clinicians with the problems caused due to resistance. Although several studies have been done to delineate AI resistance, the genetic mechanism of resistance is still not uncovered. This study was aimed at identifying the prognostic and diagnostic biomarkers for AI resistance through available data sets and computational analysis.

The breast cancer patients’ data was downloaded and differential gene expression was analyzed. It was found that 17 genes were differentially expressed, further genomic and transcriptomic analyses revealed the reason behind the dysregulation of the identified DEGs. Mutational analysis of the DEGs further narrowed down the genes that need to be focused. We found that epigenetic mechanisms are not the underlying reason behind the differential expression of the genes identified in this study. Besides, we found the involvement of the miRNA hsa-mir-1264 in regulating the expression of the *CDC20B* gene. It has been previously reported that the overexpression of CDC20 resulted in poor response in patients undergoing endocrine therapy and hence it acts as a biomarker for endocrine therapy resistance in ER-positive breast cancer patients [40]. Moreover, in our study, the *CDC20B* gene, a homolog of CDC20 was noticed to be downregulated and was also significantly mutated. Therefore, we hypothesize the role of CDC20B in acquired non-steroidal AI resistance in breast cancer patients.

Pathway analysis indicated the involvement of the gene *HSD3B1* in estrogen biosynthesis. *HSD3B1* codes for 3 beta-hydroxysteroid dehydrogenases and is responsible for catalyzing delta-5-3-beta-hydroxysteroid precursors into delta-4-ketosteroid through an oxidation reaction [41] in steroid biosynthesis. It is also responsible for the conversion of dehydroepiandrosterone (DHEA) to androstenedione in estrogen biosynthesis [42]. As delta-4-ketosteroid and androstenedione are essential for the synthesis of all steroid hormones, it can be speculated that HSD3B1 is the key factor influencing the conversion of DHEA to androstenedione. We found that the *HSD3B1* gene was upregulated in the resistant samples, indicating the increased expression of HSD3B1 in breast cancer patients on treatment with the non-steroidal AI drugs (anastrozole and letrozole). The overexpression of HSD3B1 could result in the increased production of androstenedione, which in turn elevates the estrogen hormone levels of the tumor cell. This overall mechanism would lead to the proliferation of cancer cells and pave way for non-steroidal AI resistance. The mechanism of HSD3B1 in the development of AI resistance in HR-positive breast cancer has been documented earlier [43]. These findings strengthen our prediction of HSD3B1 as a significant biomarker in AI resistance.

From the PPI network analysis, the DEGs were noticed to be highly interacting with one another. This indicates that the expression of DEGs can be influenced by one another, although further studies are required to understand the mechanism of interaction.

Additionally, it was noticed in our CNV analysis that the gene *CDKN2A* has been deleted in sensitive patients whereas it remained unaltered in resistant patients. *CDKN2A* gene is responsible for coding several proteins, especially p16, a cell division regulating protein, and a tumor suppressor gene [44]. Moreover, it was proven that the presence of mutation, differential expression, or copy number variation in the *CDKN2A* gene enhances tumor formation [45-47]. Surprisingly in our CNV analysis, *CDKN2A* gene deletion was restored in patients who are resistant to the non-steroidal AIs. Even though researchers are suggesting that CDKN2A plays a vital role in drug resistance in a variety of cancer types [48,49], its exact role and mechanism remain imprecise.

Furthermore, our findings included some upregulated genes (*MAPK4, MAPK15*, and *MAPK8IP3*) that were predicted to be mutated majorly. Previous studies have implicated the role of the MAPK pathway in AI resistance [5,20,50], but the exact mechanism behind it is still a conundrum. Studies have also shown that the increased activity of the MAPK pathway and the dysregulation of the genes involved in the pathway might contribute to the resistance of AIs [15]. Moreover, AI resistance is reported to be associated with the activation of the MAPK pathway in ER-positive breast cancer [51]. Hence, the activation of the identified DEGs (MAPK4, MAPK15, and MAPK8IP3) might trigger the MAPK pathway and thereby influence drug resistance during cancer therapy. Overall, MAPK4, MAPK15, and MAPK8IP3 may be considered significant genes in developing drug resistance toward AIs in ER-positive breast cancer patients.

The role of other DEGs (CDC42EP5, COMTD1, CRYBA4, CRYBB2, CRYBB3, CRYGS, FGFR3, GSTM21P1, RNF151, RNF208, and TP53TG5) in ER-positive cancer as well as in developing AI resistance is yet to be explored. Further *in vitro* and *in vivo* studies are required to support the involvement of CDC20B, HSD3B1, and CDKN2A, as possible prognostic biomarkers of non-steroidal AI resistance in breast cancer.

The development of drug resistance imposes a greater difficulty in the treatment of cancer and thus finding a suitable biomarker for drug resistance and proposing a feasible mechanism is crucial. In this study, we have identified a few significant genes like *CDC20B, HSD3B1, CDKN2A, MAPK4, MAPK15*, and *MAPK8IP3* that can act as potential biomarkers for non-steroidal AI resistance in ER-positive breast cancer patients. Further studies warrant a clear understanding of the mechanism behind the resistance conferred by these marker genes.

## Figures and Tables

**Table 1.** Estrogen biosynthesis pathways of the differentially expressed gene (HSD3B1) predicted using the Reactome tool

Pathway name	Entities found	Entities total	Entities p-value	Reactions found	Reactions total	Species name	Submitted entities found
Mineralocorticoid biosynthesis	1	22	0.0364	2	7	*Homo sapiens*	HSD3B1
Androgen biosynthesis	1	27	0.0444	1	9	*Homo sapiens*	HSD3B1
Glucocorticoid biosynthesis	1	29	0.0476	2	9	*Homo sapiens*	HSD3B1
Metabolism of steroid hormones	1	72	0.1143	5	40	*Homo sapiens*	HSD3B1
Metabolism of steroids	1	328	0.4277	5	244	*Homo sapiens*	HSD3B1
Metabolism of lipids	1	1444	0.9227	5	954	*Homo sapiens*	HSD3B1
Metabolism	1	3646	0.9992	5	2260	*Homo sapiens*	HSD3B1

## Supplementary Materials

Supplementary Table 1. List of differentially regulated genes involved in resistance to non-steroidal aromatase in breast cancer

Supplementary Table 2. Type of mutation present in the unique genes

Supplementary Fig. 1. Lollipop plots representing the location of the mutation in the unique genes (*TP53TG5* and *MAPK8IP3*) of the sensitive samples.

Supplementary Fig. 2. Lollipop plots were drawn for the unique genes (*CDKN2A, CRYBB2, HSD3B1*, and *MAPK15*) observed of the resistant samples.

Supplementary Fig. 3. Protein-protein interaction network of differentially regulated genes was constructed using STRING database for the visualisation possible protein-protein interaction.
