# Supplementary material for: The genomic landscape associated with resistance to aromatase inhibitors in breast cancer
Source: Genomics Inform. 2023 Jun 30;21(2):e20. doi: 10.5808/gi.23012 (PMC10326531; doi:10.5808/gi.23012)
Supplement: Supplementary Table 1. — List of differentially regulated genes involved in resistance to non-steroidal aromatase in breast cancer [file gi-23012-Supplementary-Table-1.pdf]

Supplementary Table 1. List of differentially regulated genes involved in resistance to non-steroidal aromatase in breast cancer

	Gene
Up-regulated genes	*CDC42EP5*
	CDKN2A
	COMTD1
	CRYBA4
	CRYBB2
	CRYBB3
	CRYGS
	FGFR3
	GSTM2P1
	HSD3B1
	MAPK15
	MAPK4
	MAPK8IP3
	RNF151
	RNF208
	TP53TG5
Down-regulated genes	*CDC20B*
