# Supplementary material for: The genomic landscape associated with resistance to aromatase inhibitors in breast cancer
Source: Genomics Inform. 2023 Jun 30;21(2):e20. doi: 10.5808/gi.23012 (PMC10326531; doi:10.5808/gi.23012)
Supplement: Supplementary Table 2. — Type of mutation present in the unique genes [file gi-23012-Supplementary-Table-2.pdf]

Supplementary Table 2. Type of mutation present in the unique genes

Gene name	Type of mutation
TP53TG5	Splice site mutation and missense mutation
MAPK8IP3	Missense mutation
CDKN2A	Frameshift deletion and insertion
CRYBB2	Missense mutation
HSD3B1	Missense mutation
MAPK15	Missense mutation
