# Supplementary material for: The genomic landscape associated with resistance to aromatase inhibitors in breast cancer
Source: Genomics Inform. 2023 Jun 30;21(2):e20. doi: 10.5808/gi.23012 (PMC10326531; doi:10.5808/gi.23012)
Supplement: Supplementary Fig. 1. — Lollipop plots representing the location of the mutation in the unique genes (TP53TG5 and MAPK8IP3) of the sensitive samples. [file gi-23012-Supplementary-Fig-1.pdf]

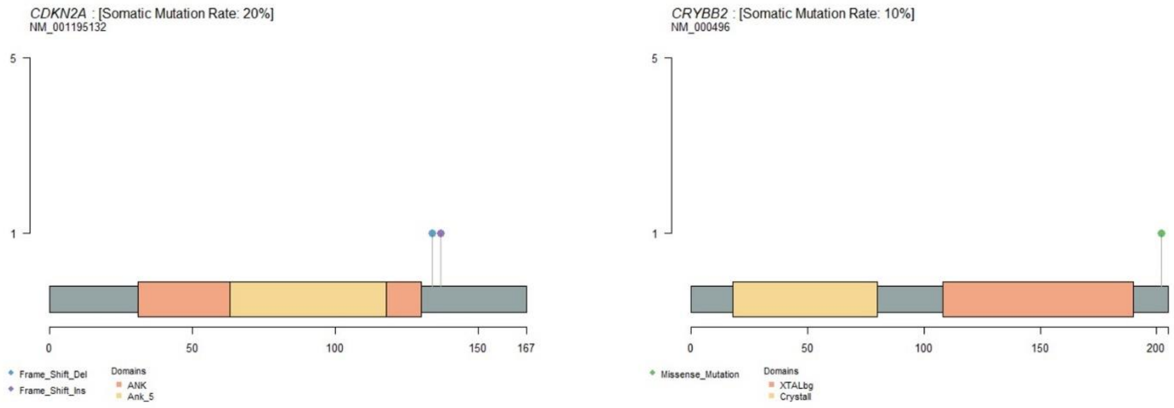

(a) (b)

Supplementary Fig. 1. Lollipop plots representing the location of the mutation in the unique genes (*TP53TG5* and *MAPK8IP3*) of the sensitive samples.
