# Supplementary material for: The genomic landscape associated with resistance to aromatase inhibitors in breast cancer
Source: Genomics Inform. 2023 Jun 30;21(2):e20. doi: 10.5808/gi.23012 (PMC10326531; doi:10.5808/gi.23012)
Supplement: Supplementary Fig. 2. — Lollipop plots were drawn for the unique genes (CDKN2A, CRYBB2, HSD3B1, and MAPK15) observed of the resistant samples. [file gi-23012-Supplementary-Fig-2.pdf]

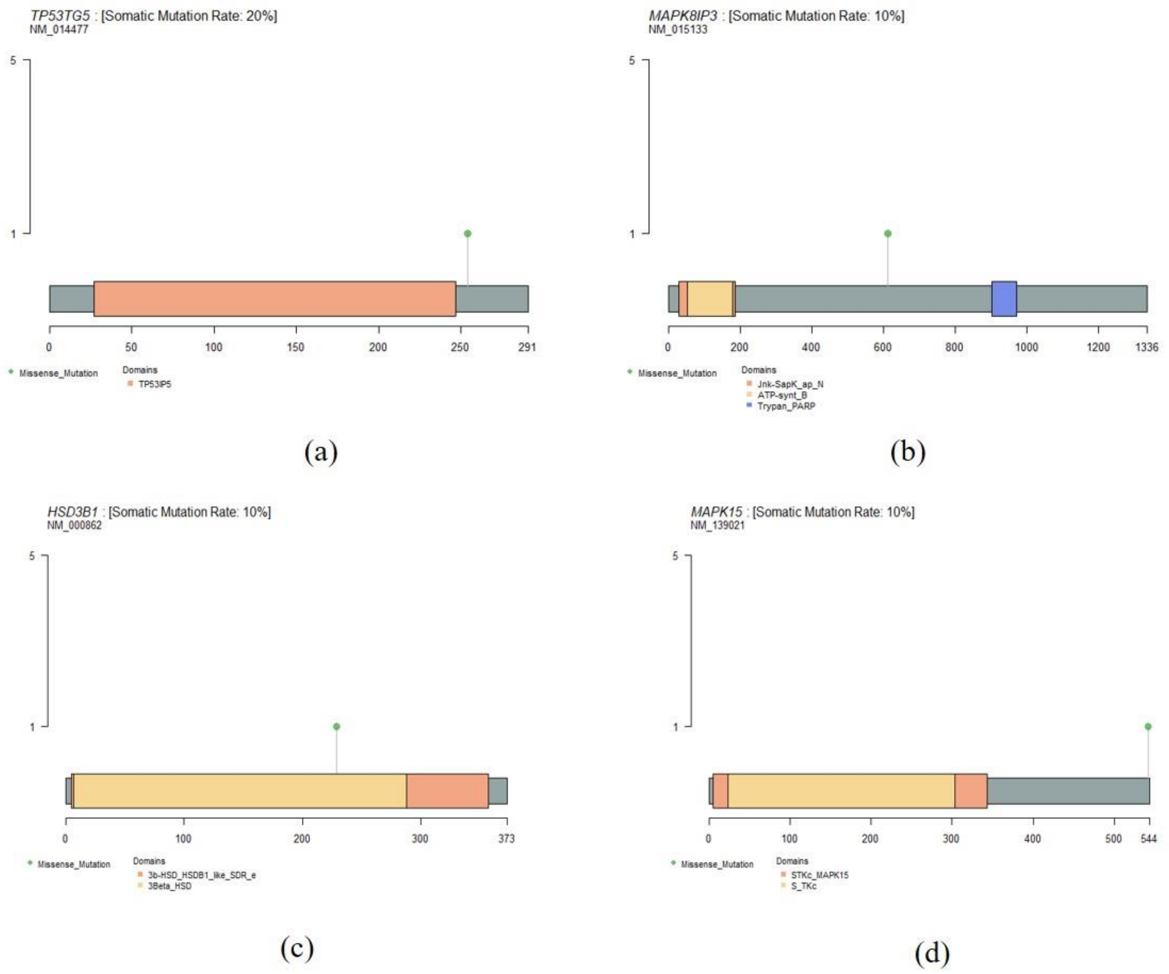

Supplementary Fig. 2. Lollipop plots were drawn for the unique genes (*CDKN2A*, *CRYBB2*, *HSD3B1*, and *MAPK15*) observed of the resistant samples.
