# Supplementary material for: The genomic landscape associated with resistance to aromatase inhibitors in breast cancer
Source: Genomics Inform. 2023 Jun 30;21(2):e20. doi: 10.5808/gi.23012 (PMC10326531; doi:10.5808/gi.23012)
Supplement: Supplementary Fig. 3. — Protein-protein interaction network of differentially regulated genes was constructed using STRING database for the visualisation possible protein-protein interaction. [file gi-23012-Supplementary-Fig-3.pdf]

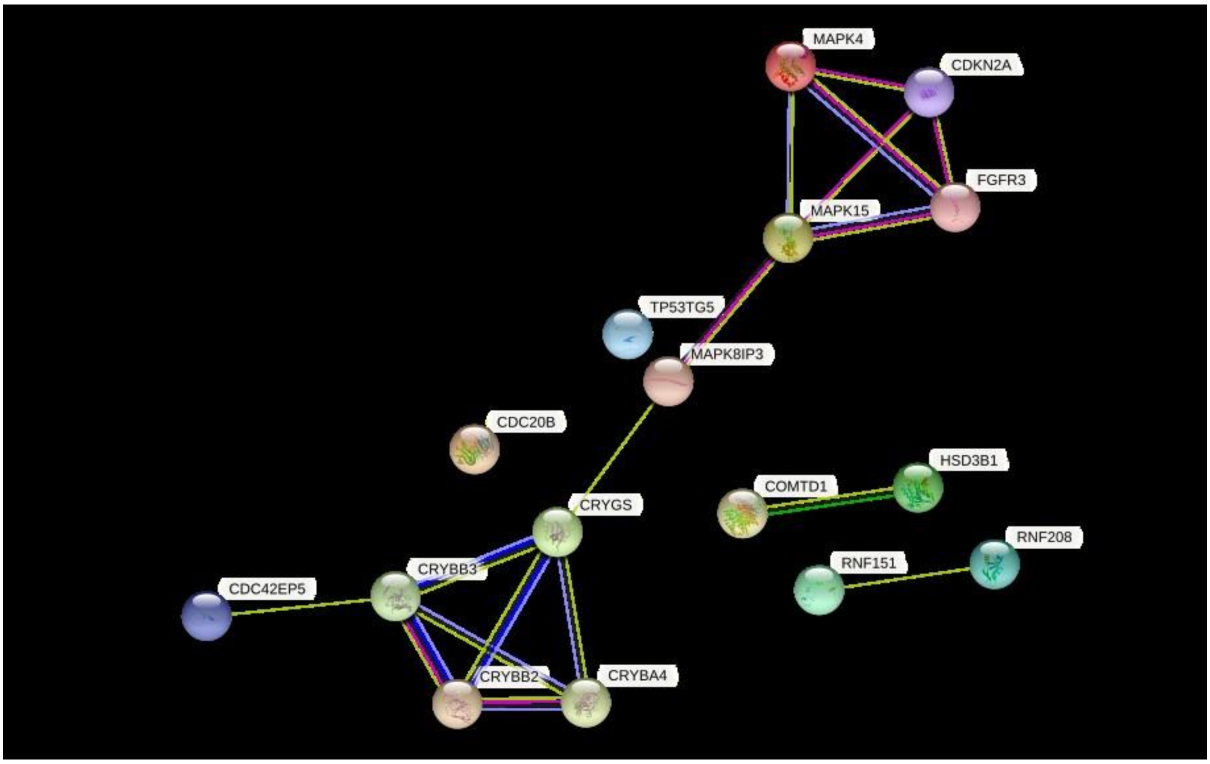

Supplementary Fig. 3. Protein-protein interaction network of differentially regulated genes was constructed using STRING database for the visualisation possible protein-protein interaction.
